# Melkersson-Rosenthal Syndrome: a Case Report of the Classic Triad

**DOI:** 10.30476/DENTJODS.2019.77804.

**Published:** 2020-12

**Authors:** Molook Torabi, Mehrnaz Karimi Afshar, Hoda Barati

**Affiliations:** 1 Kerman Social Determinants on Oral Health Research Center, Dept. of Oral Pathology, School of Dentistry, Kerman University of Medical Sciences, Kerman, Iran; 2 Resident, Dept. of Prosthodontics, School of Dentistry, Tehran University of Medical Sciences, Tehran, Iran; 3 Resident, Dept. of Oral Medicine, School of Dentistry, Tehran University of Medical Sciences, Tehran, Iran

**Keywords:** Melkersson-Rosenthal syndrome, Case report, Classic type

## Abstract

Melkersson-Rosenthal syndrome (MRS) is a rare neurological condition that includes a triad of symptoms including recurring facial paralysis, orofacial swelling, and fissured tongue. The diagnosis and treatment of this syndrome is difficult since the classic triad is rarely possible to see in its complete form. The etiology of MRS is unknown, but it is thought to be caused by various factors such as infections, genetic predisposition, immune deficiency, food intolerance, and stress. This case report presents a 22- year-old male patient with classical triad of MRS

## Introduction

Melkersson-Rosenthal syndrome (MRS) is a rare disorder that is manifested by the clinical triad of recurring facial nerve paralysis, swelling of one or both lips (cheilitis granulomatosa), and fissural tongue [ 1 - 3 ]. MRS begins in childhood or adolescence [ 4 ]. This syndrome may appear alternately after the first time. After frequent attacks (from days to years), edema may remain, increase, and finally become permanent [ 5 ]. The etiology of MRS is unknown but there might be a genetic condition [ 1 , 5 ]. 

The classic triad is rarely seen completely, therefore, the diagnosis of this syndrome is difficult [ 1 , 6 ]. The most common and first symptom of MRS is orofacial swelling. The fissured tongue is seen in about 20-40% of the affected individuals and may have been present since birth; facial paralysis occurs in about 30% of patients [ 7 ]. In histopathologic features of a classic form of cheilitis granulomatosa edema and dilation of lymphatic vessels in superficial lamina propria, and scattered aggregates of noncaseating granulomatous inflammation, consisting of lymphocytes and histiocytes are present [ 8 ]. In this report, a patient with all aspects of the classical triad is presented.

## Case Report

**Figure 1:**
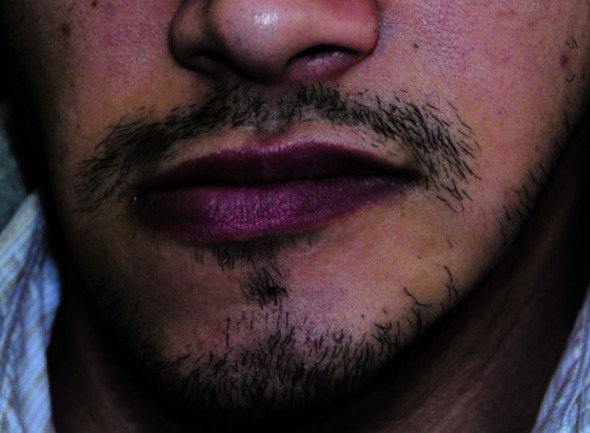
Swelling and asymmetry in the right side of the lower lip and face of the patient

**Figure 2:**
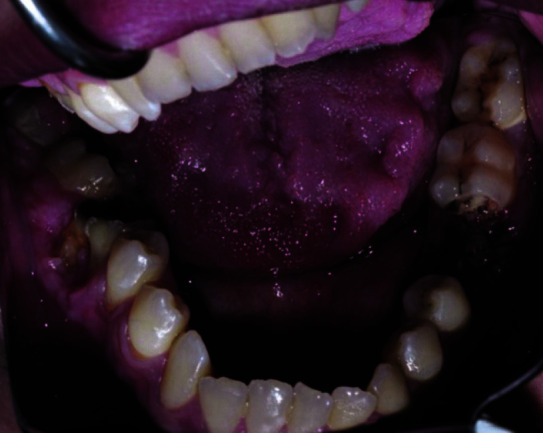
Fissured tongue

A 22- year-old man referred to Prosthodontics Department, Dental School, Tehran Medical Sciences University for receiving prosthetic treatments. He had swelling of the right side of the lower lip, and paralysis of face (Figure 1). He reported that the edema of his lip has been repeatedly occurred with certain intervals. In addition, patient’s history showed that he had suffered from facial paralysis and facial edema in right side of his face for many years. On intraoral examination, it was observed that he had macroglossia and deep fissures on the dorsal and lateral surfaces of the tongue (Figure 2). 

There were no history of systemic diseases, regular drug use, and adenopathy. Patient was consulted with the Oral Medicine Department, School of Dentistry, Tehran Medical Sciences University and he was diagnosed with MRS based on the clinical findings (complete triad of signs).

## Discussion

MRS is a rare neuro-mucocutaneous disorder with a recurrent and progressive course, and characterized by the triad of lip swelling, fissured tongue, and facial paralysis. The most common clinical presentation are oligosymptomatic or monosymptomatic forms [ 9 ]. 

The classic triad of symptoms of MRS is seen in only 8% to 18% of patients and the mono-symptomatic or oligo-symptomatic form is more common. This turns diagnosis of this disease to be very challenging [ 9 - 10 ]. In this case, all three clinical findings were observed at the time of referral to the clinic. Therefore, he was diagnosed with MRS based on clinical symptoms.

The first manifestation of MRS is usually edema of upper lip, lower lip, one or both lips, eyelids, or rarely one side of the scalp. Lips (especially upper lip) are involved more frequently [ 1 ]. In our patient, swelling was seen on the right side of the lower lip. In addition, fissures were seen on the right side of the dorsal of tongue. The third finding in our patient was facial paralysis, which was present for many years. 

In patients affected, swelling has a tendency for recurrence and usually completely subsides in the early stages [ 12 - 13 ] but it can last longer and become a fixed enlargement of orofacial tissues. Therefore, early diagnosis and appropriate treatment can prevent future undesired complications [ 5 ]. 

This patient was 22 years old. Elias and et al. [ 9 ] in a study on 75 cases with MRS, reported the age range of 8-9 years. Feng and et al. [ 14 ] in a study on 44 cases reported the age onset to be 14 years. However, diagnosis of many involved patients may be delayed for several decades [ 9 ].

The etiology of MRS is not clear yet. It seems that MRS is due to genetic factors in some cases because it involves several members of their families. Other factors as dietary, allergens, infections (especially herpes simplex virus), immune deficiency, and stress may also be involved [ 4 , 15 ]. Therefore, treatment of this rare disease is difficult [ 4 ] and symptomatic; it may include treatment with nonsteroidal anti-inflammatory agents, corticosteroids, antibiotics, and anti-depressant drugs [ 5 , 16 - 17 ]. Surgery may be prescribed to reduce the pressure on the facial nerves and reduce swelling, but its effectiveness has not been proven [ 18 - 19 ]. Regardless of the type of treatment, patients should be regularly examined even if they have no clinical symptoms since the course of MRS is chronically progressive [ 5 ].

Our patient was referred to the Oral Medicine Department and oral corticosteroid therapy was administered for him. Dental impressions were taken for the diagnosis and treatment plan concerning his prosthetic treatments and by the time of this report, he is completing his pre prosthetic treatments.

Inform consent and ethical permission was taken from the patient for publishing his medical documents.

## Conclusion

The orofacial swelling of the MRS can be prolonged and become permanent. Hence, early detection and proper treatment can prevent unwanted side effects. Dentists should consider MRS for patients with recurrent swelling in the lips, and they should refer them to the relevant specialists. 
